# Treatment Trajectories of Young People With Emotion Dysregulation Engaging in Dysregulated Behaviours: An Exploratory Study

**DOI:** 10.1192/bjo.2024.462

**Published:** 2024-08-01

**Authors:** Bibire Baykeens, Martina Di Simplicio

**Affiliations:** 1 Imperial College London, London, United Kingdom; 2 West London NHS Trust, London, United Kingdom

## Abstract

**Aims:**

Dysregulated behaviours are prevalent amongst young people worldwide. Emotional dysregulation plays a key role in these behaviours. Several psychiatric disorders have significant elements of emotional dysregulation, making it a potentially effective transdiagnostic therapeutic target. Dialectical behaviour therapy (DBT), mentalisation-based therapy (MBT), and schema therapy (ST) can effectively manage emotional dysregulation, however access may be limited in clinical practice.

We aimed to explore whether young people with emotional dysregulation engaging in dysregulated behaviours receive support for emotion regulation. We hypothesised that those with emotionally unstable personality disorder (EUPD) will have a higher prevalence of self-harm, disordered eating, and/or substance misuse and more referrals for DBT, MBT, or ST than those with bipolar disorder, autism, attention deficit/hyperactivity disorder (ADHD), schizophrenia, or schizoaffective disorder.

**Methods:**

De-identified clinical records from the West London NHS Trust on 2,413 16- to 25-year-olds with an ICD–10 diagnosis of EUPD, bipolar disorder, autism, ADHD, schizophrenia, and/or schizoaffective disorder were obtained through Akrivia. Chi-squared tests were performed.

**Results:**

Young people with bipolar disorder had the highest prevalence of self-harm, disordered eating, and substance misuse (88.35%, n = 182), χ2 (4, N = 3138) = 39.14, p < 0.001, but the lowest number of references to DBT, MBT, or ST. Those with EUPD had the highest number of references to DBT, χ2 (4, N = 2585) = 886.75, p < 0.001, MBT, χ2 (4, N = 2585) = 81.63, p < 0.001, or ST, LR (4, N = 2585) = 21.03, p < 0.001.

**Conclusion:**

There could be an unmet need for psychological interventions for young people with bipolar disorder. A more transdiagnostic approach to offering psychotherapies that target emotional dysregulation should be applied in clinical practice.

